# Reply to: Re-evaluating evidence for adaptive mutation rate variation

**DOI:** 10.1038/s41586-023-06315-x

**Published:** 2023-07-26

**Authors:** J. Grey Monroe, Kevin D. Murray, Wenfei Xian, Thanvi Srikant, Pablo Carbonell-Bejerano, Claude Becker, Mariele Lensink, Moises Exposito-Alonso, Marie Klein, Julia Hildebrandt, Manuela Neumann, Daniel Kliebenstein, Mao-Lun Weng, Eric Imbert, Jon Ågren, Matthew T. Rutter, Charles B. Fenster, Detlef Weigel

**Affiliations:** 1 University of California Davis, Davis, CA USA; 2 Max Planck Institute for Biology Tübingen, Tübingen, Germany; 3 Department of Plant Biology, Carnegie Institution for Science, Stanford, CA USA; 4 Department of Biology, Stanford University, Stanford, CA USA; 5 Department of Biology, Westfield State University, Westfield, MA USA; 6 ISEM, University of Montpellier, Montpellier, France; 7 Department of Ecology and Genetics, EBC, Uppsala University, Uppsala, Sweden; 8 Department of Biology, College of Charleston, Charleston, SC USA; 9 Oak Lake Field Station, South Dakota State University, Brookings, SD USA

**Keywords:** Evolution, Genetics

replying to L. Wang et al. *Nature* 10.1038/s41586-023-06314-y (2023)

Wang and colleagues^1^ argue that our report^2^ of lower mutation rates in gene bodies, essential genes and regions marked by H3K4me1 must result from DNA sequencing errors. We appreciate the issues raised by them and by other colleagues^3^. Although we overlooked some sources of errors, these are insufficient to invalidate our conclusions, which are confirmed by more stringent reanalyses of our original data, new analyses restricted to high-confidence germline mutations^4^, and direct demonstration of plant DNA repair proteins being recruited to gene bodies, essential genes and H3K4me1, where they reduce local mutation rates^5,6^.

Wang and colleagues^1^ identify issues with somatic mutation calling, suggesting that homopolymer bleed-through errors in Illumina sequencing are responsible for patterns observed in somatic mutations, and that elevated cytosine deamination in transposable elements is responsible for the patterns in germline mutations. Here we address these concerns.

Consecutive runs of identical nucleotides, or homopolymers, pose challenges to discovering rare mutations because they can lead to Illumina sequencing errors at immediately neighbouring nucleotides through homopolymer bleed-through^7^. At the same time, homopolymer regions have higher true mutation rates even at local but non-adjacent sites (for example, ref. ^8^). Wang and colleagues^1^ found that the distribution of simulated homopolymer errors mirrors the overall distribution of mutations we reported around genes (their Fig. 1a). However, there are several reasons why such homopolymer errors cannot be the source of inferred mutation bias.

**Fig. 1:**
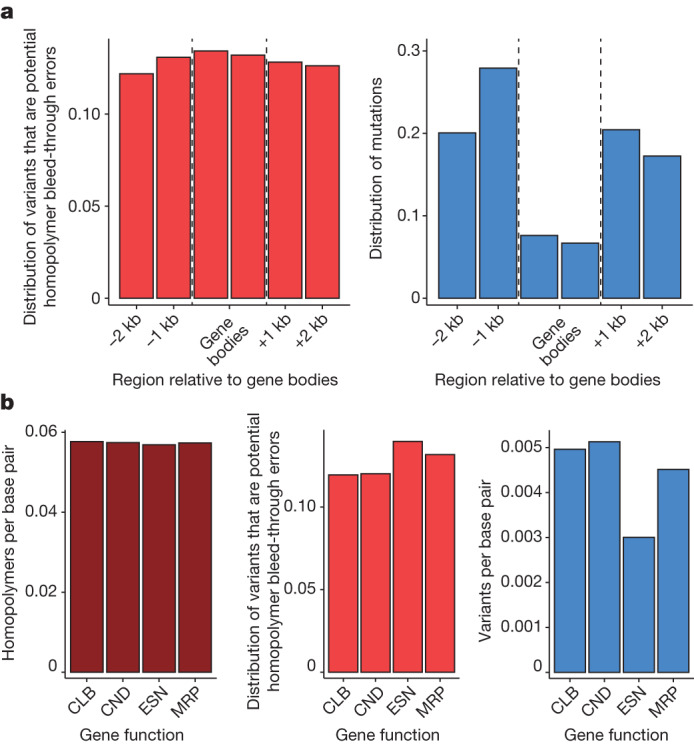
Potential homopolymer bleed-through sequencing errors cannot explain differences in mutation rate. **a**, The proportion of variants that are potential homopolymer bleed-through errors among all mutation calls in our original study^2^ is as least as high in gene bodies as in intergenic sequences, and contrasts with the distribution of total mutation calls. kb, kilobase. **b**, Homopolymers and the proportion of variants that are potential homopolymer bleed-through errors in the original study^2^ are not lower in essential genes (ESN) compared to genes with environmentally conditional (CND), morphological (MRP) and cellular or biochemical (CLB) functions, and cannot explain the distribution of actual mutation calls.

Wang and colleagues^1^ assume that homopolymer bleed-through errors affect sequences up to five nucleotides away from homopolymers, although these errors occur on modern Illumina platforms at positions immediately adjacent to a run of identical bases^7^. Moreover, their simulation of sequencing errors apparently assumes that 100% of sequencing errors occur as a product of homopolymer bleed-through. By contrast, empirical estimates of sequencing errors report only 0.7 to 5.2% to be the result of homopolymer bleed-through^7^. Across all data in our study, only 12.0% of total single-nucleotide variant calls (10.2% for high-quality germline calls) could be potential homopolymer-adjacent bleed-through errors, and thus on their own cannot explain the approximately 50% mutation rate reduction we observed in gene bodies relative to intergenic regions^2^.

More importantly, Wang and colleagues’ own analysis^1^ reports that the proportion of potential homopolymer bleed-through errors in our data is actually higher in gene bodies (exons plus introns), which should lead to gene body mutation rates being overestimated, not underestimated. We confirm across our datasets that the proportion of potential homopolymer bleed-through errors is not lower in gene bodies (Fig. 1a, left), and differs from the pattern of mutation calls (Fig. 1a, right). Similarly, the proportion of potential homopolymer bleed-through errors is not reduced in essential genes (Fig. 1b). The distribution of potential homopolymer bleed-through errors, therefore, disagrees with the hypothesis of Wang and colleagues^1^. By contrast, the observed pattern is expected if gene bodies and essential genes experienced a reduction in true mutation rates, as noise introduced by sequencing errors should have a proportionally larger effect on regions with truly low mutation rates.

Homopolymeric sequences (but not potential homopolymer bleed-errors) are enriched outside gene bodies, as reported by Wang and colleagues^1^. Thus, the observed mutation rate heterogeneity is consistent with previous evidence that homopolymer-rich regions have higher true mutation rates^8^ and that their enrichment in these regions is consistent with the expected long-term evolutionary consequence of lower DNA repair activity, as the expansion of homopolymers is a signature of lower mismatch repair activity (Supplementary Note 3). Moreover, both preferential repair of exons by mismatch repair and higher intronic mutation rates in somatic tissues have been widely documented (Supplementary Note 3). Likewise, considerable differences in mutation rate and spectra between somatic and germline cells are well known, with somatic cells having orders of magnitude higher mutation rates. Indeed, differences between mitotic and meiotic cells have been previously proposed for *Arabidopsis thaliana* by Wang and colleagues^9^ (Supplementary Note 3).

Wang and colleagues^1^ further suggest that the patterns we observed in germline mutations might result largely from elevated deamination of methylated cytosines (GC-to-AT mutations) in transposable elements. Several findings are inconsistent with this hypothesis: cytosine methylation was included as a covariate in our original models, mutation accumulation experiments consistently indicate that mutation rates are lower in gene bodies relative to non-transposable element intergenic regions in *A. thaliana* (Fig. 2a,b; see below), and removing all GC-to-AT mutations from our original germline dataset does not alter the observed pattern, with H3K4me1 remaining the strongest epigenomic predictor of lower mutation (described in detail recently^4^). The same has been demonstrated for mutation rate variation in rice, in which mutation rates are lower in gene bodies relative to both intergenic regions and transposable elements^6^.

**Fig. 2:**
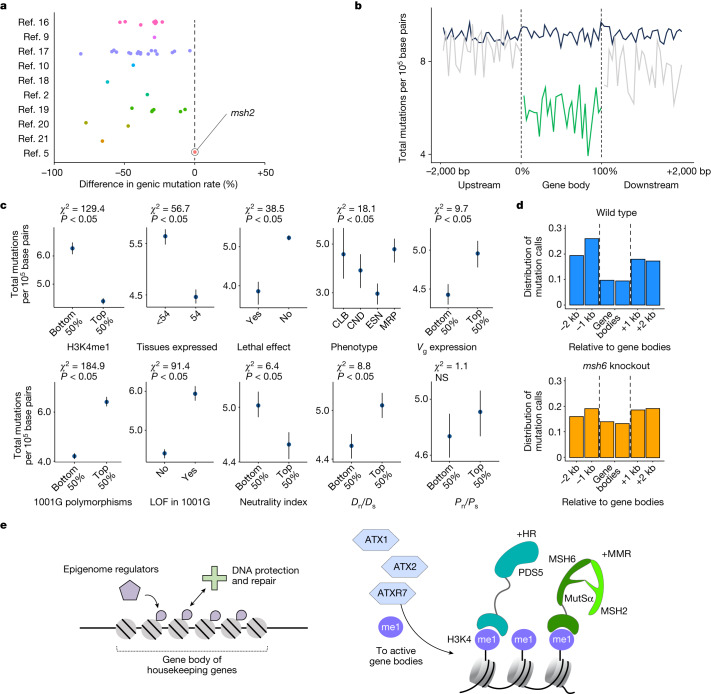
Joint analyses of germline mutations in several published *A. thaliana* mutation accumulation studies align with mechanistic models of mutation bias. **a**, Reduction in genic single-nucleotide germline mutation rates compared against genomic background in multiple *A. thaliana* datasets (Supplementary Table 1). For our original study^2^, only new mutations from 400 mutation accumulation lines are shown; the other mutations in that paper were already described^10^ and are shown separately here. Mutation rate reduction in genic regions is eliminated in *msh2* DNA repair mutants^5^. bp, base pair. **b**, Mutation rates around gene bodies (grey and green lines). Black line indicates randomly selected windows based on gene lengths. **c**, Mutation rates in genes classified by functional category, rates of sequence evolution, patterns of expression and estimates of selection. Significance tested with *χ*^2^ test, *n* = 27,206 genes, with raw *P* values tested against *α* = 0.05 (unadjusted for multiple comparisons). Data show mean values for groups ± error bars reflecting 95% confidence intervals from bootstrapping. *V*_g_, genetic variance of gene expression; 1001G, 1001 Genomes Project; LOF, loss of function; *D*_n_, non-synonymous divergence; *D*_s_, synonymous divergence; *P*_n_, non-synonymous polymorphism; *P*_s_, synonymous polymorphism; NS, not significant. **d**, Somatic mutations identified with very stringent criteria and using a caller specifically designed for rare somatic mutations, Strelka2, are reduced in gene bodies of wild-type plants, but not *msh6* mutants^6^. **e**, Left, general mechanism proposed in ref. ^2^. Right, new knowledge regarding biochemical mechanisms underlying reduced mutation rates in gene bodies established by recent discoveries in plants and synthesized in ref. ^6^. HR, homology-directed repair; MMR, mismatch repair^17–22^.

To further address concerns with somatic mutation calls in general, we re-called putative somatic mutations in the original 107 lines^10^ by mapping reads to an improved reference genome^11^ and applying more stringent filtering (Supplementary Note 1). This led to more complete and higher-quality read mapping (Supplementary Fig. 1) and resolved several issues described by Wang and colleagues^1^ (for example, high intron-versus-exon mutation ratio and the proportion of potential homopolymer bleed-through errors; Supplementary Fig. 2). These data confirm that gene bodies experience lower mutation rates, including when manually removing potential homopolymer bleed-through errors (Supplementary Note 1). Many of the analyses by Wang and colleagues are affected by unreliable centromeric mutations, which constituted 41% of questioned somatic mutations^1^. These sites, however, could not have affected our conclusions because they were excluded from all of our original analyses (Supplementary Note 2 and Supplementary Fig. 3).

Wang and colleagues^1^ examined essential genes with approaches that were not in our original study. They used subsets of our initial datasets, focusing on either about 2,000 germline or about 4,000 somatic single-nucleotide variants, finding that neither dataset directly revealed a statistically significantly lower mutation rate in essential genes. This approach seems underpowered, yielding near-zero values for mutation counts in entire gene classes, an indication that the data are poorly suited for *χ*^2^ approximation (Supplementary Note 5).

In our study^2^, we had instead modelled genome-wide mutation rates, and using these models, identified a connection between gene essentiality and mutation rate corresponding to epigenome differences—essential genes are enriched for H3K4me1, for example, which we found to be associated with lower mutation rates. We subsequently tested whether this expectation is met in a very large set of several hundred thousand loosely filtered putative somatic mutations with ample power to compare gene classes. We agree that somatic mutation calling is very difficult, as most real somatic mutations and unrepaired damaged sites (with DNA damage occurring 10,000 to 100,000 times per day per cell; Supplementary Note 3) are expected to be present in only one cell and thus detectable only by a single read. In Supplementary Note 4 and an accompanying Correction^12^, we discuss why singletons were called as putative mutations in one of our reanalyses, from 64 leaves^13^, owing to inadvertently mapping forward reads twice. However, analyses of variant quality in these data do not support the hypothesis that our results are simply due to higher rates of poor-quality calls in non-genic regions or non-essential genes (Supplementary Note 4 and Supplementary Fig. 4).

Finally, to directly address the possibility that our conclusions reflect unknown sources of bias in inherently uncertain somatic calls, we reanalysed germline mutations from our study^2^ along with mutation accumulation experiment data generated in several independent studies (Supplementary Table 1). This meta-analysis of >10,000 germline mutations confirmed the previously reported, nearly universal reduction in single-nucleotide mutation rates in gene bodies, essential genes and regions marked by H3K4me1 (Fig. 2a–c; ref. ^4^). The notable exception comes from plants lacking the mismatch repair protein MSH2 (Fig. 2a; ref. ^5^). A similar pattern is seen when somatic mutations were called with very stringent criteria in plants deficient for the MSH2 partner MSH6, using a tool specifically designed for rare somatic mutations^14^ (Fig. 2d). This was as predicted from H3K4me1 directly attracting MSH6 to gene bodies^6^, confirming that DNA repair in *A. thaliana* is targeted to gene bodies, as is well known in humans (Supplementary Note 3). Finally, analyses of >43,000 experimentally induced de novo germline mutations in rice (previously validated with 99% accuracy) also show that gene bodies, conserved genes, and H3K4me1-marked regions experience lower mutation rates, even when considering only silent (synonymous) mutations^6^.

Relationships between histone modifications, DNA repair, and mutation rate are widely known (Supplementary Note 3). Our work^2^ considered the evolutionary implication of these relationships. We had leveraged models of the drift-barrier hypothesis to discover that natural selection could favour mechanisms linking DNA repair to widely distributed epigenomic features, such as H3K4me1, which is not only enriched in gene bodies and essential genes in *A. thaliana*, but also the histone modification most strongly associated with lower mutation rates in our data^2^. An important higher-order test of our conclusions is therefore whether they are mechanistically supported. Since publication of our article^2^, it has been demonstrated that plant DNA repair proteins are recruited by H3K4me1 to gene bodies and essential genes. These repair proteins, which contain Tudor ‘reader’ domains that bind H3K4me1, include PDS5C, involved in homology-directed repair, and MSH6, which functions as a dimer with MSH2 in the mismatch repair pathway and recruits MutY of the base-excision repair pathway^15^. The genome-wide distribution of PDS5C, as measured by chromatin immunoprecipitation followed by sequencing^4,6,16^, confirms that regions subject to elevated repair protein activity coincide with features at which we detected lower spontaneous mutation rates^4,6,16^.

We conclude that the reported relationships between epigenomic features and mutation rates^2^ are well supported mechanistically (Fig. 2e). We agree that there are issues and inherent uncertainties with somatic mutation calling, which make it difficult to know the accuracy of individual calls in the very large set of loosely filtered somatic variants^2^. However, the proposal that the observed patterns result only from sequencing errors is inconsistent with multiple lines of evidence from the original study, independent analyses and emerging parallel work.

## Reporting summary

Further information on research design is available in the Nature Portfolio Reporting Summary linked to this article.

## Online content

Any methods, additional references, Nature Portfolio reporting summaries, source data, extended data, supplementary information, acknowledgements, peer review information; details of author contributions and competing interests; and statements of data and code availability are available at 10.1038/s41586-023-06315-x.

## Supplementary information

Supplementary Information This file contains Supplementary Table 1, Notes 1–5 (with Figs 1–4) and References.

Reporting Summary

## Data Availability

The TAIR10 *A. thaliana* reference genome can be found at https://arabidopsis.org/download. The more recent, improved *A. thaliana* reference genome can be found at https://github.com/schatzlab/Col-CEN. Sequencing reads for 107 *A. thaliana* mutation accumulation lines are stored in the National Center for Biotechnology Information Short Read Archive, accession number SRP133100. Additional mutation datasets were downloaded from publications cited in Supplementary Table 1.

## Code availability

Code for this work uses functions maintained in https://github.com/greymonroe/polymorphology, with additional scripts and data for analyses and figures in https://github.com/greymonroe/mutation_bias_analysis2.
