# Supplementary material for: Reply to: Re-evaluating evidence for adaptive mutation rate variation
Source: Nature. 2023 Jul 26;619(7971):E57–60. doi: 10.1038/s41586-023-06315-x (PMC10371858; doi:10.1038/s41586-023-06315-x)
Supplement: Supplementary file 1 — This file contains Supplementary Table 1, Notes 1–5 (with Figs 1–4) and References. [file 41586_2023_6315_MOESM1_ESM.pdf]

nature portfolio

https://doi.org/10.1038/s41586-023-06315-x

Supplementary information

Reply to: Re-evaluating evidence for adaptive mutation rate variation

In the format provided by the authors and unedited

Supplemental Table 1. Relative genic germline mutation rate (only single-base-pair substitutions) in multiple data sets.

Proportion of mutations in genic regions	Δ from expectations based on genome content of UTRs + coding seq + introns	Number of mutations	Genotype	Additional information	Treatment	Reference
0.50	+0.002	4,048	*msh2* mutant		Untreated	[1]
0.17	-0.66	92	Col-0 wild type		Heat	[2]
0.36	-0.29	31	Col-0 wild type (D)	Lines	Untreated	[3]
0.35	-0.30	69	Col-0 wild type (E)	Lines	Warming	[3]
0.45	-0.10	54	Col-0 wild type (F)	Lines	Heat	[3]
0.35	-0.30	60	Col-0 wild type (A)	Population	Untreated	[3]
0.21	-0.45	130	Col-0 wild type (B)	Population	Warming	[3]
0.47	-0.07	88	Col-0 wild type (C)	Population	Heat	[3]
0.28	-0.44	1,865	Col-0 wild type		Untreated	[4]
0.36	-0.27	121	*nrpe* mutant		Untreated	[5]
0.31	-0.38	107	*ddc* mutant		Untreated	[5]
0.24	-0.53	98	Col-0 wild type		Untreated	[5]
0.25	-0.49	83	Col wild type		Salt 125	[5]
0.36	-0.29	197	*nrpe* mutant		Salt 125	[5]
0.36	-0.28	255	*ddc* mutant		Salt 125	[5]
0.36	-0.28	244	*ddc* mutant		High Salt 150	[5]
0.39	-0.23	252	*ddc* mutant		High Salt 175	[5]
0.19	-0.62	99	Col-0 wild type		Untreated	[6]
0.33	-0.34	1,685	Non Col-0 wild type	Germline	Untreated	[7]
0.26	-0.47	72	Col-0 wild type		Saline	[8]
0.11	-0.77	44	Col-0 wild type		Untreated	[8]
0.20	-0.59	32	Col-0 wild type		Untreated	[9]
0.22	-0.56	9	Col-0 wild type		UV 150	[9]
0.31	-0.39	13	Col-0 wild type		UV 230	[9]
0.30	-0.40	38	Col-0 wild type		UV 300	[9]

0.10	-0.81	21	*uvr8* mutant	Untreated	[9]
0.27	-0.46	33	*uvr8* mutant	UV 300	[9]
0.42	-0.16	19	*tt4* mutant	Untreated	[9]
0.31	-0.39	23	*tt4* mutant	UV 300	[9]
0.27	-0.47	89	*uvh1* mutant	Untreated	[9]
0.21	-0.57	120	*uvh1* mutant	UV 300	[9]
0.31	-0.39	12	*uvr2* mutant	Untreated	[9]
0.34	-0.33	181	*uvr2* mutant	UV 300	[9]
0.43	-0.14	13	*uvr3* mutant	Untreated	[9]
0.48	-0.03	29	*uvr3* mutant	UV 300	[9]
0.30	-0.41	25	*uvr2 uvr3* mutant	Untreated	[9]
0.35	-0.31	153	*uvr2 uvr3* mutant	UV 150	[9]
0.37	-0.27	279	*uvr2 uvr3* mutant	UV 230	[9]
0.36	-0.28	1,409	*uvr2 uvr3* mutant	UV 300	[9]
0.36	-0.29	316	Col-0 x Ler hybrid progeny	Untreated	[10]

Supplemental Note 1

To address concerns about erroneous calls in the data used for building our models of mutation rate and epigenomic features, we re-mapped trimmed reads (to remove low quality calls at the end of reads) from the 107 *Arabidopsis* mutation accumulation lines originally published in ref. [4] to a more complete and accurate reference genome in *A. thaliana*[11]. Distribution of mapping quality and completeness confirm improvements from using this new reference (Fig. S1). We called variants from mapped reads using GATK HaplotypeCaller, as in the original study[7].

We re-analyzed our data according to the filter suggested by Wang and colleagues[12], retaining only variants with fewer than 10 samples having any read with a specific non-reference (ALT) call, and applied also additional filters: ALT base quality >30; only 1 sample having reads with the ALT call on both strands, and only 1 ALT variant observed in that sample; total depth of Q20 reads between 11 and 99; and 100% (or <90%) of reads supporting the ALT call for putative germline (or somatic) mutations (Fig. S2).

These reanalyses confirmed the significant reduction of gene body mutation rates as originally reported. This was true when we filtered to only include variants with at least 2 reads supporting the ALT call on both strands in a sample. The quality of variant bases was high and similar for germline and somatic mutations (~39). The intron/exon ratio was similar for putative somatic (0.4-0.6) and germline (0.4) mutations, and it was much lower than for the originally reported somatic mutations (>5), a focal point of concern from Wang and colleagues. The fraction of clustered variants (<10 bp apart) was less than 7%, and removal of such variants had no obvious effect on results. The filtered variants do not support the hypothesis that reduced gene body mutation rates is the effect of homopolymer bleed-through errors. Only ~10-13% of variants are potential homopolymer bleed-through errors (neighboring homopolymer repeats >2 bp long), and their removal does not have a substantial impact on the results. See Fig. S2 for more details.

To benchmark these results, we asked how they compare with other data sources. The consistent observations of lower gene body mutation rates in our original and reanalyzed set of variants align with a large body of external support: (i) widespread evidence from all mutation accumulation experiments conducted in *A. thaliana* (Fig. 2a in the main text and ref. [13]), (ii) high-confidence somatic mutations in *A. thaliana* (Fig. 2d in the main text and ref. [14]), (iii) induced mutations in rice[14], (iv) empirical distribution of DNA repair protein activity across the *A. thaliana* genome[14–16], (v) enrichment of gene bodies for H3K4me1, a known target of DNA repair[14,15], (vi) experimentally confirmed preferential repair of gene bodies in *A. thaliana*[1,14], and (vii) population genetic signatures of mutation rate variation in *A. thaliana*[7].

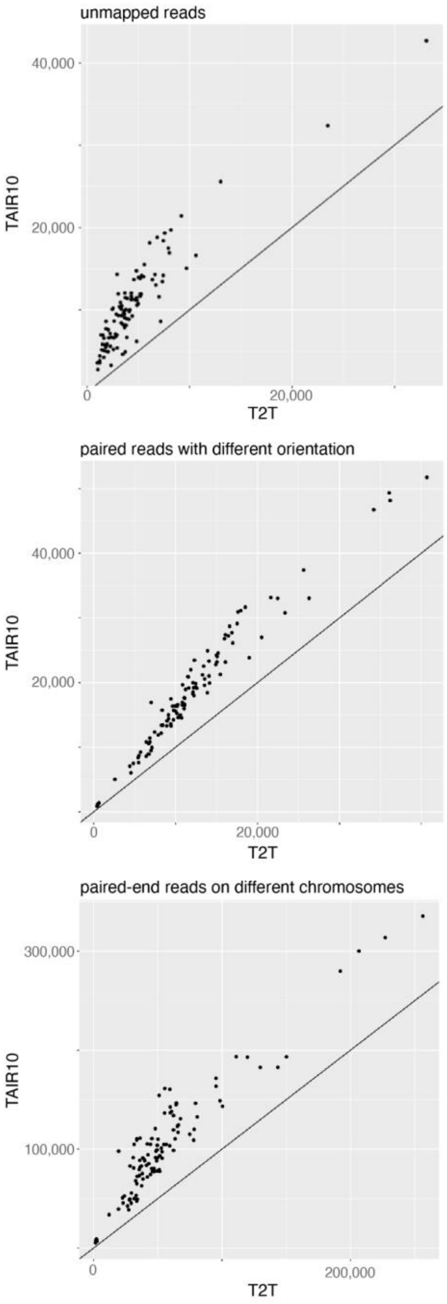

Figure S1. Improvements in mapping completeness and quality by using a telomere-to-telomere reference genome. Individual points represent summary data from each of the 107 mutation accumulation lines. T2T on the X-axis refers to reads mapped against an *A. thaliana* Col-0 telomere-to-telomere reference genome[11], TAIR10 on the Y-axis refers to reads mapped against the original, less complete TAIR10 reference genome. The line indicates unity. Values are consistently above this line, indicating that mapping metrics in all samples are improved.

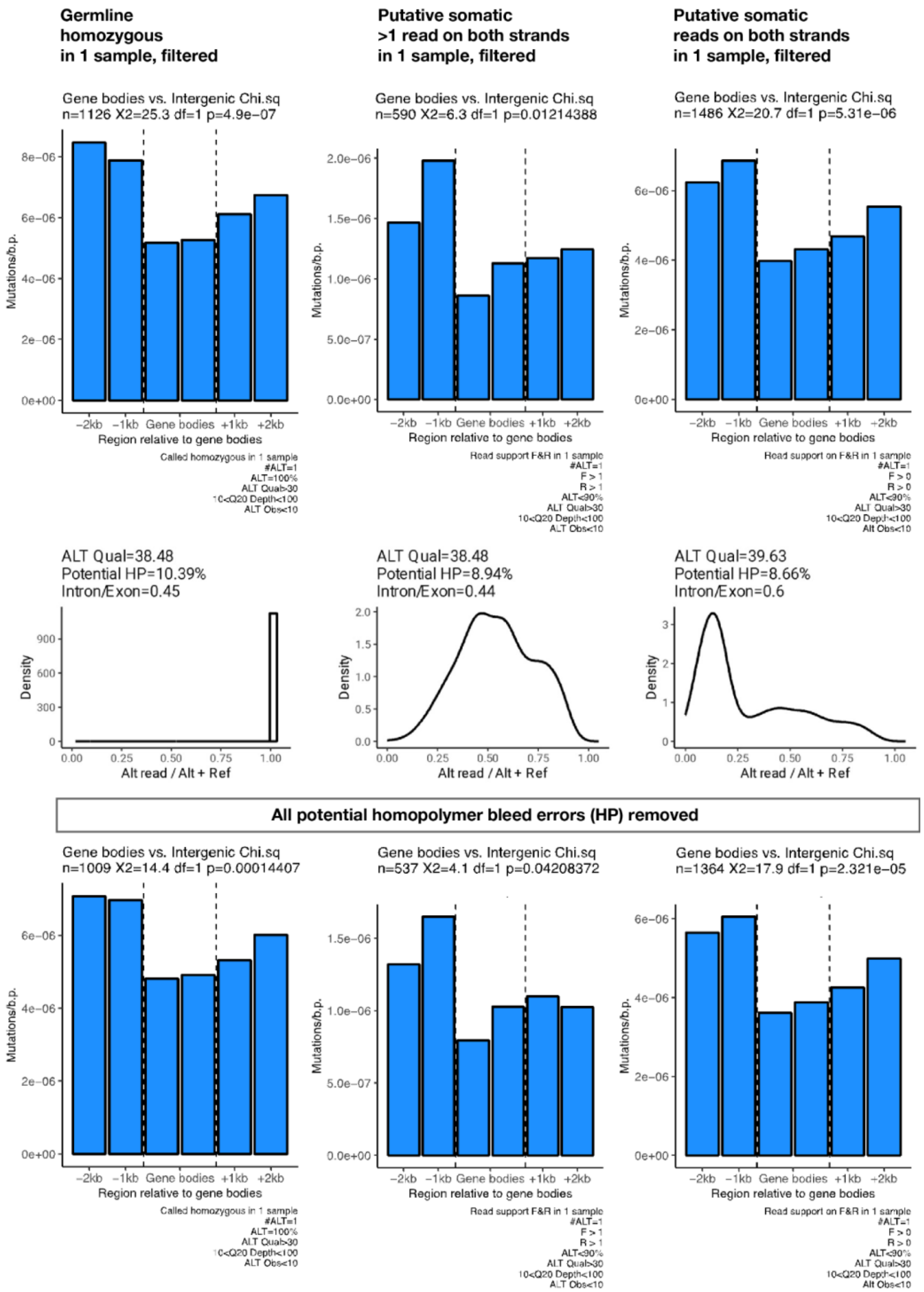

Figure S2. Remapped variants against new reference genome with improved filters. Variants were identified from 107 mutation accumulation lines[4]. Top panels show estimated mutation rates in and around gene bodies based on variants obtained with different filters. The total number of variants in the filtered dataset is given as *n*, additional details below each panel. The χ^2 tests are based on a 2x2 contingency table comparing variant counts and sequence space for gene bodies vs. neighboring (±2 kb) sequences. The panels in the middle show corresponding variant allele frequency distributions (ALT/ALT+ REF). ALT quality, potential homopolymer bleed-through error (HP), and intron/exon variant ratios are shown as well. The bottom row shows mutation rates around genes after removing potential

homopolymer sites.

Supplemental Note 2

Wang and colleagues[12] highlight in their Fig. 1e a large number of centromeric variants on chromosomes 3, 4, and 5, and they conclude that our analyses must have been affected by these unreliable mutations in our original set of more stringently filtered somatic mutation calls from a previously published data set[4] (corresponding to 41% of the variants Wang and colleagues[12] call in question). However, our focus was specifically on mutations within 2 kb of gene bodies and models of epigenome features affecting mutation rates around genes. Pericentromeric regions are gene poor, and therefore few, if any, variants that result from mismapping at repetitive sequences around centromeres were considered in our analyses (Fig. S3).

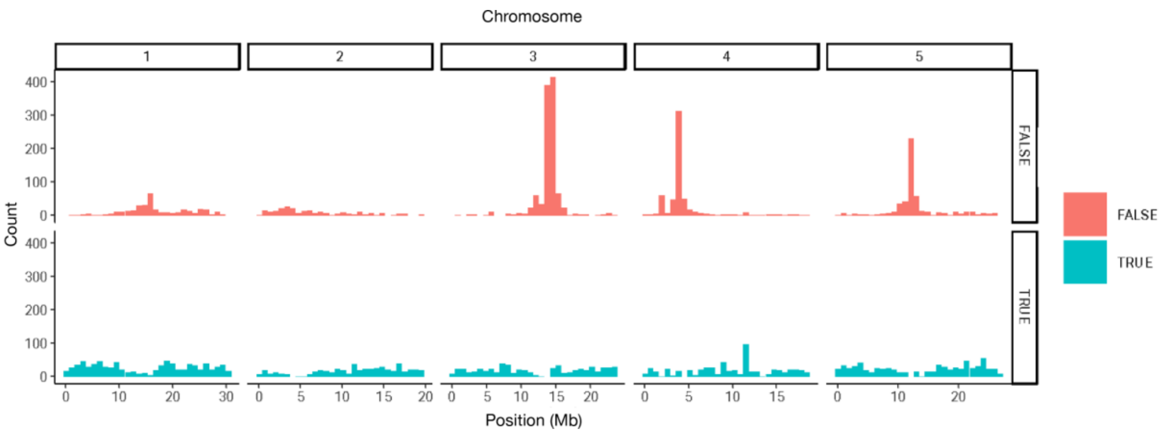

Figure S3. Potentially problematic mutation calls near centromeres were *de facto* excluded from analyses in our paper to study mutation rates and epigenomic features around genes. Shown here are the variants that were used (TRUE) vs. excluded (FALSE) to produce models that could predict associations between mutation rates and epigenomic features.

Supplemental Note 3

EXTENDED DISCUSSION

Somatic versus germline mutations

The central critique of Wang and colleagues[12] is based on their analysis of differences between germline and somatic mutations in our data. In the main text of our Reply, we tested the proposal of Wang and colleagues[12] that the pattern of somatic mutations we reported could be explained by sequencing errors. We find that sequencing errors alone cannot explain our results. For a more complete discussion, here we consider the analyses and interpretation of Wang and colleagues[12] in light of what is known about mutation rates and patterns in somatic versus germline cells in different organisms.

Mutation rates are often found to be orders of magnitude greater in somatic than in germline cells[17–20]. This is thought to be driven largely by the lower activity of several DNA repair pathways in somatic cells, especially in cells that are mitotically inactive (as is the case for terminally differentiated leaf cells in plants, for example)[18,21–23]. While the extent to which plants maintain a fully and truly segregated germline remains an area of ongoing research[24,25], a growing body of evidence suggests that rates and patterns of somatic and germline somatic mutations are distinct in plants, with DNA repair being considerably more active in meristems, which harbor the pluripotent cells that will eventually go on to contribute to the germline in reproductive organs[25–30].

In mammals, somatic mutation rates (mutations/unit time) scale negatively with life span, with short-lived species having orders of magnitude higher somatic mutation rates[20]. Whether this is true in plants as well has yet to be thoroughly tested, though the per-year mutation rate in annuals such as *Arabidopsis thaliana* appears to be orders of magnitude higher than in long-lived perennials[31,32]. With DNA damage occurring on the order of 10,000 to 100,000 times per day in every cell[33–36], relatively small differences in DNA repair activity in somatic tissue between short and long-lived species could readily lead to considerable differences in observed mutation rates.

Apart from the global mutation rate, the spectrum (e.g., frequency of mutations in specific trimer contexts) and distribution (e.g., exon versus intron) of mutation can vary considerably between somatic tissues and between soma and germ. Intron mutation rates are generally higher relative to exon mutation rates in somatic tissue[1,19,37–40]. The spectrum of mutation is ultimately due to biological factors, driven by the balance between sources of DNA damage/errors in replication and DNA repair at different types of sites. Different ‘clock-like mutational signatures’ and frequencies of specific base-pair changes relate to both differences in prevailing sources of DNA damage such as UV stress and differences in repair activity between cell types, species, and genotypes[19,20,41–43]. It may therefore be ill-advised to interpret simple differences in mutation spectra between samples as *de facto* evidence of sequencing errors without understanding and considering other sources of variation. Because most of our understanding of mutation signatures are derived from studies of human cancer and germline mutations, more work is

needed to elucidate the underlying causes of differences in mutation spectra among tissues in plants.

Relationships between epigenome, DNA repair, and mutation rates

Wang and colleagues[12] argue that relationships between epigenomic features (i.e., histone modifications) and mutation rates observed in our study are likely unsound. To give broader context to this claim, it is important to consider the current understanding of the causes of mutation rate variation. The role of epigenomic features in driving patterns of mutation rate variation across genomes is among the most well-established phenomena in mutation rate research[37,39,40,44–54]. For example, in vertebrates, PWWP domains in DNA repair proteins bind H3K36me3, which marks exons of actively expressed genes, thereby increasing rates of repair and lowering mutation rates in regions enriched for H3K36me3. Disruption of H3K36me3, for example through inactivation of SETD2, the histone methyltransferase responsible for establishing H3K36me3 at transcribed exons, is a hallmark of numerous cancers[37,55,56].

In plants, emerging discoveries indicate that DNA repair proteins contain Tudor domains, which can apparently be recruited to H3K4me1, which marks actively expressed gene bodies in a plant-specific pattern[14–16]. This observation is consistent with lower mutation rates in H3K4me1 marked regions, namely gene bodies of transcriptionally active genes, in plants[7,14,57], or the finding that the efficiency of CRISPR-mediated mutagenesis is lower in H3K4me1-marked regions, indicative of more efficient repair[5,58,59]. Taking a broad view, such discoveries suggest a remarkable example of convergent evolution between vertebrates – which have evolved PWWP domains in repair proteins that target H3K36me3 in active genes – and plants – which have evolved Tudor domains in repair proteins that target H3K4me1 in active genes.

Distribution of homopolymer sequences

An interesting observation made by Wang and colleagues[12] is that the distribution of homopolymer regions mirrors the distribution of observed mutations in our data. They thus hypothesized that the distribution of reported mutations was primarily driven by homopolymer bleed-through errors. We find that such errors cannot explain the pattern: consistent with the analysis of Wang and colleagues[12], we find that likely bleed-through errors are proportionally more frequent in gene bodies, as expected if the true mutation rate in those regions is low.

The distribution of homopolymers in the *A. thaliana* genome may provide interesting insight into mutation rate variation. Homopolymer-rich regions are known to have high mutation rates[60–65], and their distribution may be a consequence of elevated mutation rate, as homopolymers arise themselves from lower mismatch repair activity. Whether the distribution of homopolymer sequences along genomes can be used to study the long-term consequences of mutation rate heterogeneity is unclear, but it could provide a deeper understanding of how differential DNA repair shapes broad patterns of genome and sequence architecture.

Supplemental Note 4

Discussion of implications of accompanying Correction

We agree that care is needed to eliminate false positives in somatic mutation calling. In our original manuscript, we aimed to test whether mutation distributions observed in original mutation accumulation experiments in *A. thaliana*. To this end, we re-analyzed a number of independent datasets. For only one of these data sets (64 deeply sequenced leaves[66]), we inadvertently mapped reads twice to the genome, potentially leading to an excess of singletons being called. We address this read mapping error with an accompanying Correction[67] and discuss its implications in the following. To assess the possibility that singleton variants introduced a source of error that could explain the distribution of variants called in this dataset[66], we have now performed a series of tests to examine the distribution of signatures of error in these data along the genome. We find that only 10.0% of variants called were potential homopolymer bleed-through errors, a known source of error[68]. Variant quality scores (QD) indicate a greater proportion of low-quality sequencing variants in gene bodies and essential genes, which is the opposite of what is expected if sequencing errors explained the lower mutation rates in gene bodies and essential genes. Variants had been filtered in our original analysis[7] to include only those called in a single sample to remove sites with recurrent error. Still, it is impossible to distinguish unrepaired DNA damage and mutations in single cells from singleton sequencing errors in the data. In a recent preprint[13], we explored how different filters affected the reported results[7], finding that more stringent filtering of the original data had minimal effects on our conclusions[13].

In the work leading to our article[7], we had explored different filtering parameters for somatic mutations. Because we found qualitatively similar patterns in the combined analyses of germline and somatic mutations, we maximized the number of mutations in several summary figures by using very loose filtering criteria. Specifically, we first used 2,257 germline and 6,317 stringently filtered somatic mutation calls (single-nucleotide substitutions and small indels) based on the initial study of 107 mutation accumulation lines[4] to build a model of epigenome-driven mutation rate bias. For the empirical confirmation of reduced mutation rates in gene bodies and essential genes, we added 8,891 germline mutation calls from a natural mutation accumulation line experiment[69], 3,306 germline and 355,827 loosely filtered somatic mutation calls from 400 additional mutation accumulation lines first reported in our article, and 773,141 somatic mutation calls from 64 leaves[66] (which included a large number of inadvertently called singletons, as described in the Correction of our original article[7]). Singleton calls in these data actually tended to have higher base quality (QD=7.5 vs 3.7, $t = -264.38$, p-value $< 2.2e-16$), and they were less likely to be potential homopolymer bleed-through errors (8.7 % vs 14.4%, $\chi^2 = 2693.4$, p-value $<$ 2.2e-16) compared to variant calls supported by multiple reads in these data, which is consistent with the expectation that real DNA damage or mutations should be found in only a single sample, whereas low-quality sequencing errors, such as homopolymer bleed-through errors, are more likely to arise recurrently and therefore to be represented by multiple independent reads.

The original study reporting sequencing data from 64 leaves[66] identified a much smaller number

of somatic mutations, only 16 single-nucleotide variants, because it prioritized true positives over false negatives by only including very high-frequency somatic mutations that likely arose early during development and are thus shared by a large number of cells. The vast majority of somatic mutations, however, will only be found in a single or in very few cells, and most somatic mutations will therefore be very difficult to detect. Importantly, in our variant calls from the 64-leaves data set, we found that variant calls in gene bodies did not have higher per-base quality or mapping quality than adjacent intergenic regions. Differences in per-base quality or mapping quality, therefore, can not explain the observed pattern of reduced variant calls in gene bodies (Fig. S4).

To further assess whether the distribution of these variants is likely to be grounded in biology, we can compare ultra-high quality mutation datasets. We have recently identified somatic mutations in *A. thaliana* plants sequenced at 250X depth and analyzed with Strelka2, a stringent cancer mutation caller[70]. This analysis confirmed that the distribution of our original somatic mutation calls is consistent with the patterns observed with very high-quality somatic mutations[14] (Fig. 2d in the main text of this Reply). We nevertheless acknowledge that somatic mutation data require careful consideration. Future somatic mutation calling in plants should leverage deep sequencing and cancer mutation calling methods, instead of population calling methods such as the GATK Haplotypecaller used in our study[7].

In plants, we do not know the real somatic mutation rate per cellular division or per chronological time in non-dividing tissue, or how it differs between meristematic and differentiated cells. DNA is damaged 10,000 to 100,000 times per day in each cell, which can be detectable in sequencing data[33–36,71,72]. Instances of unrepaired damage, if not corrected before or during replication, go on to become transmissible mutations. An analysis of human somatic mutations in clonal tissue found 484,678 single base pair mutations in 389 healthy samples sequenced at 27x coverage[19], and another study reported up to ~7 mutations/Mb in healthy human cells, from samples of approximately 1,000 cells obtained by microdissection[73].

A study in the annual plant *Cannabis sativa* reported 816,835 mosaic mutations in a single individual[74]. Based on empirical measurements and by extrapolating from relationships between life history and somatic mutation rates, somatic mutation rates in *A. thaliana* could be many orders of magnitude higher than in humans[20,75]. Because we did not sequence individual somatic cells, it is difficult to estimate their DNA damage/mutation rate, but a back-of-the-envelope calculation of ~12,000 sequenced genomes in the 400 additional mutation accumulation lines would yield a rough estimate of a lower bound of 3×10^{-7} bp^{-1} per somatic cell. A similar calculation would yield a rough estimate of 10^{-5} bp^{-1} per somatic cell for the 64-leaves data set, with the proviso that these calls included singletons and it is possible that a large fraction of sites with unrepaired damage (for example, oxidized guanines) were included. Similar to mutations, the distribution of DNA damage that has not yet been repaired should reflect local differences in the efficiency of the DNA repair machinery. Thus, even if many singleton calls correspond to unrepaired DNA damage, we would expect them to be reduced in regions such as gene bodies and essential genes that experience elevated repair, for example, regions where DNA damage is being constantly repaired through mechanisms that are linked to

transcription as well as regions enriched for H3K4me1 (ref. [76,77]).

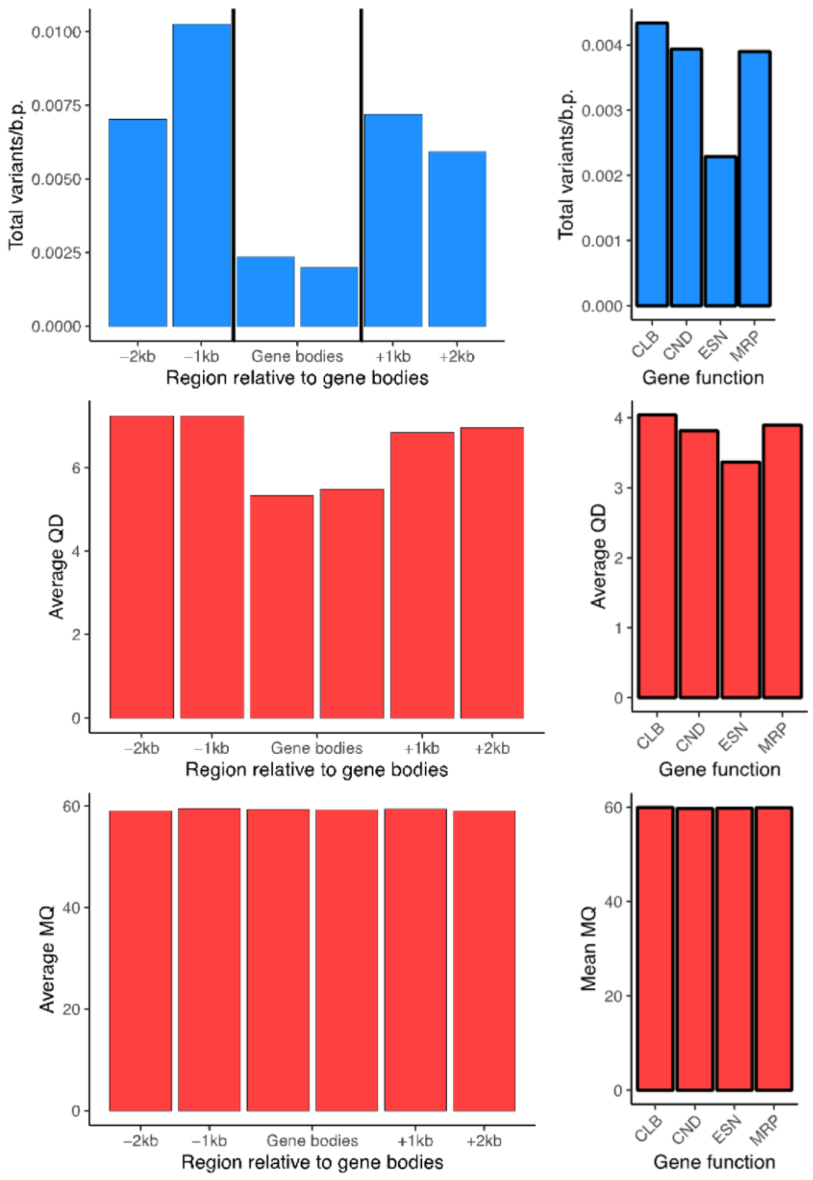

Figure S4. Evaluating potential evidence of low-quality calls affecting the identification of putative somatic mutations from 64 deeply sequenced leaves. The base quality (QD) is lower in gene bodies and essential genes, which does not support the proposal that higher mutation rates in non-genic and non-essential genes are due to elevated sequencing error in these regions. Over 95% of variants had a mapping quality (MQ) of 60 (the maximum possible). Essential genes (ESN) are compared to genes with environmentally conditional (CND), morphological (MRP), and cellular/biochemical (CLB) functions.

Supplemental Note 5

χ^2 tests

In a related preprint that we published last year[13], we had applied χ^2 tests to a highly filtered dataset to address potential concerns that mutation rates in intergenic regions are not higher than in gene bodies, and instead caused by C>T mutations in transposable elements, which are enriched in intergenic regions. We agree that such highly filtered datasets are not ideal for comparisons of gene classes, e.g., essential versus non-essential genes, with χ^2 tests because very stringent filtering results in near-zero counts for entire classes of genes. χ^2 tests are thus not only underpowered, but such data sets are also poorly-suited for accurate χ^2 approximation because they violate assumptions for χ^2 tests.

Here, we report the more straightforward analysis from the complete datasets of >10,000 germline mutations identified across multiple studies (Supplemental Table 1). These analyses confirm the observed lower essential gene mutation rates at P-value <<<0.01. In our original paper[7], we reported the difference between essential and other genes across the entire gene body, including UTRs, and observed an approximately 30% reduction, which we therefore re-tested (and confirmed) in the large germline mutation dataset (Figure 2a-c of the main text).

Variant re-filtering

Mapping quality and completeness were both improved by using a higher quality reference (Figure S1). We called variants using GATK HaplotypeCaller, which reports informative reads only and uses a base quality threshold of 10. In the previous submission, we used these reported reads to identify variants that were called in multiple samples. Reads not reported by GATK were therefore not included in our filtering. We have now re-analyzed these data (raw reads from the 107 *A. thaliana* mutation accumulation lines originally published in ref. [4]) in light of the comments of Wang and colleagues[12] and the filtering methods they suggested. We re-analyzed our data based on variants with <10 samples containing ALT reads and applied additional filters: ALT base quality > 30, variants where only 1 sample contained reads supporting on both strands and in which only 1 ALT variant was observed in that sample, a total depth of Q20 reads greater than 10 and less than 100, and 100% percent of reads supporting the ALT for putative germline mutations and <90% for putative somatic mutations.

These re-analyses confirm the significant reduction of gene body mutation rates as originally reported. This was true when we filtered to only include variants with 2 or more reads supporting the ALT call on both strands in a sample. The mean base quality of variants was high and the same for germline and somatic mutations (~39). The intron/exon ratio is similar between somatic (0.4-0.6) and germline (0.4) calls and much lower than originally reported (>5) for somatic mutations, which was a focal point of concern from Wang and colleagues[12]. The fraction of clustered variants (<=10 bp apart) was less than 7%, and the removal of such variants had no effect on our results. Finally, the filtered data still do not support the hypothesis

that the inference of reduced gene body mutation rates reflects the effect of homopolymer bleed-through errors. Only ~10-13% of variants are potential homopolymer bleed-through errors (neighboring repeats of >2 bp) in these data, and their removal does not have a substantial impact on the results. See Figure S2 in the paper for details.

Supplemental References

1. Belfield, E. J. *et al.* DNA mismatch repair preferentially protects genes from mutation. *Genome Res.* **28**, 66–74 (2018).
2. Belfield, E. J. *et al.* Thermal stress accelerates Arabidopsis thaliana mutation rate. *Genome Res.* **31**, 40–50 (2021).
3. Lu, Z. *et al.* Genome-wide DNA mutations in Arabidopsis plants after multigenerational exposure to high temperatures. *Genome Biol.* **22**, 160 (2021).
4. Weng, M.-L. *et al.* Fine-Grained Analysis of Spontaneous Mutation Spectrum and Frequency in Arabidopsis thaliana. *Genetics* **211**, 703–714 (2019).
5. Zhu, X. *et al.* Non-CG DNA methylation-deficiency mutations enhance mutagenesis rates during salt adaptation in cultured Arabidopsis cells. *Stress Biology* **1**, 12 (2021).
6. Ossowski, S. *et al.* The rate and molecular spectrum of spontaneous mutations in Arabidopsis thaliana. *Science* **327**, 92–94 (2010).
7. Monroe, J. G. *et al.* Mutation bias reflects natural selection in Arabidopsis thaliana. *Nature* (2022) doi:10.1038/s41586-021-04269-6.
8. Jiang, C. *et al.* Environmentally responsive genome-wide accumulation of de novo Arabidopsis thaliana mutations and epimutations. *Genome Res.* **24**, 1821–1829 (2014).
9. Willing, E.-M. *et al.* UVR2 ensures transgenerational genome stability under simulated natural UV-B in Arabidopsis thaliana. *Nat. Commun.* **7**, 13522 (2016).
10. Yang, S. *et al.* Parent-progeny sequencing indicates higher mutation rates in heterozygotes. *Nature* **523**, 463–467 (2015).
11. Naish, M. *et al.* The genetic and epigenetic landscape of the *Arabidopsis* centromeres.

Science **374**, eabi7489 (2021).

12. Wang, L., Ho, A. T., Hurst, L. D., Yang, S. Re-evaluating evidence for adaptive mutation rate variation. *Nature* https://doi.org/10.1038/s41586-023-06314-y (2023).
13. Monroe, J. G. *et al.* Report of mutation biases mirroring selection in Arabidopsis thaliana unlikely to be entirely due to variant calling errors. *bioRxiv* 2022.08.21.504682 (2022) doi:10.1101/2022.08.21.504682.
14. Quiroz, D. *et al.* Biased mutagenesis and H3K4me1-targeted DNA repair in plants. *bioRxiv* 2022.05.28.493846 (2022) doi:10.1101/2022.05.28.493846.
15. Niu, Q. *et al.* A histone H3K4me1-specific binding protein is required for siRNA accumulation and DNA methylation at a subset of loci targeted by RNA-directed DNA methylation. *Nat. Commun.* **12**, 3367 (2021).
16. Pradillo, M. *et al.* Involvement of the Cohesin Cofactor PDS5 (SPO76) During Meiosis and DNA Repair in Arabidopsis thaliana. *Front. Plant Sci.* **6**, 1034 (2015).
17. Gorbunova, V., Seluanov, A., Mao, Z. & Hine, C. Changes in DNA repair during aging. *Nucleic Acids Res.* **35**, 7466–7474 (2007).
18. Bujarrabal-Dueso, A. *et al.* The DREAM complex functions as conserved master regulator of somatic DNA-repair capacities. *Nat. Struct. Mol. Biol.* (2023) doi:10.1038/s41594-023-00942-8.
19. Moore, L. *et al.* The mutational landscape of human somatic and germline cells. *Nature* (2021) doi:10.1038/s41586-021-03822-7.
20. Cagan, A. *et al.* Somatic mutation rates scale with lifespan across mammals. *Nature* **604**, 517–524 (2022).
21. Lans, H. *et al.* Involvement of global genome repair, transcription coupled repair, and chromatin remodeling in UV DNA damage response changes during development. *PLoS Genet.* **6**, e1000941 (2010).
22. Mueller, M. M. *et al.* DAF-16/FOXO and EGL-27/GATA promote developmental growth in

response to persistent somatic DNA damage. *Nat. Cell Biol.* **16**, 1168–1179 (2014).

23. Vermezovic, J., Stergiou, L., Hengartner, M. O. & d'Adda di Fagagna, F. Differential regulation of DNA damage response activation between somatic and germline cells in Caenorhabditis elegans. *Cell Death Differ.* **19**, 1847–1855 (2012).
24. Lanfear, R. Do plants have a segregated germline? *PLoS Biol.* **16**, e2005439 (2018).
25. Watson, J. M. *et al.* Germline replications and somatic mutation accumulation are independent of vegetative life span in Arabidopsis. *Proc. Natl. Acad. Sci. U. S. A.* **113**, 12226–12231 (2016).
26. Velappan, Y., Signorelli, S. & Considine, M. J. Cell cycle arrest in plants: what distinguishes quiescence, dormancy and differentiated G1? *Ann. Bot.* **120**, 495–509 (2017).
27. Kimura, S. *et al.* DNA repair in higher plants; photoreactivation is the major DNA repair pathway in non-proliferating cells while excision repair (nucleotide excision repair and base excision repair) is active in proliferating cells. *Nucleic Acids Res.* **32**, 2760–2767 (2004).
28. Cruz-Ramírez, A. *et al.* A SCARECROW-RETINOBLASTOMA protein network controls protective quiescence in the Arabidopsis root stem cell organizer. *PLoS Biol.* **11**, e1001724 (2013).
29. Satterlee, J. W., Strable, J. & Scanlon, M. J. Plant stem-cell organization and differentiation at single-cell resolution. *Proc. Natl. Acad. Sci. U. S. A.* **117**, 33689–33699 (2020).
30. Uchiyama, Y., Kimura, S., Yamamoto, T., Ishibashi, T. & Sakaguchi, K. Plant DNA polymerase lambda, a DNA repair enzyme that functions in plant meristematic and meiotic tissues. *Eur. J. Biochem.* **271**, 2799–2807 (2004).
31. Schmid-Siegert, E. *et al.* Low number of fixed somatic mutations in a long-lived oak tree. *Nat Plants* **3**, 926–929 (2017).
32. Perez-Roman, E., Borredá, C., López-García Usach, A. & Talon, M. Single-nucleotide mosaicism in citrus: Estimations of somatic mutation rates and total number of variants. *Plant Genome* **15**, e20162 (2022).

33. Lodish, H. *et al.* Molecular Biology of the Cell. Preprint at (2008).

34. Manova, V. & Gruszka, D. DNA damage and repair in plants - from models to crops. *Front. Plant Sci.* **6**, 885 (2015).

35. Yousefzadeh, M. *et al.* DNA damage—how and why we age? *Elife* **10**, e62852 (2021).

36. Nisa, M.-U., Huang, Y., Benhamed, M. & Raynaud, C. The Plant DNA Damage Response: Signaling Pathways Leading to Growth Inhibition and Putative Role in Response to Stress Conditions. *Front. Plant Sci.* **10**, 653 (2019).

37. Li, F. *et al.* The histone mark H3K36me3 regulates human DNA mismatch repair through its interaction with MutSα. *Cell* **153**, 590–600 (2013).

38. Supek, F. & Lehner, B. Clustered Mutation Signatures Reveal that Error-Prone DNA Repair Targets Mutations to Active Genes. *Cell* **170**, 534–547.e23 (2017).

39. Huang, Y., Gu, L. & Li, G.-M. H3K36me3-mediated mismatch repair preferentially protects actively transcribed genes from mutation. *J. Biol. Chem.* **293**, 7811–7823 (2018).

40. Sun, Z. *et al.* H3K36me3, message from chromatin to DNA damage repair. *Cell Biosci.* **10**, 9 (2020).

41. Alexandrov, L. B. *et al.* The repertoire of mutational signatures in human cancer. *Nature* **578**, 94–101 (2020).

42. Jiang, P. *et al.* A modified fluctuation assay reveals a natural mutator phenotype that drives mutation spectrum variation within Saccharomyces cerevisiae. *Elife* **10**, (2021).

43. Sasani, T. A. *et al.* A natural mutator allele shapes mutation spectrum variation in mice. *Nature* **605**, 497–502 (2022).

44. Habig, M., Lorrain, C., Feurtey, A., Komluski, J. & Stukenbrock, E. H. Epigenetic modifications affect the rate of spontaneous mutations in a pathogenic fungus. *Nat. Commun.* **12**, 5869 (2021).

45. de la Peña, M. V., Summanen, P. A. M., Liukkonen, M. & Kronholm, I. Chromatin structure influences rate and spectrum of spontaneous mutations in Neurospora crassa. *bioRxiv*

2022.03.13.484164 (2022) doi:10.1101/2022.03.13.484164.

46. Yang, X. *et al.* Developmental and temporal characteristics of clonal sperm mosaicism. *Cell* **184**, 4772–4783.e15 (2021).
47. Yan, W., Deng, X. W., Yang, C. & Tang, X. The Genome-Wide EMS Mutagenesis Bias Correlates With Sequence Context and Chromatin Structure in Rice. *Front. Plant Sci.* **12**, 579675 (2021).
48. Davarinejad, H. *et al.* The histone H3.1 variant regulates TONSOKU-mediated DNA repair during replication. *Science* **375**, 1281–1286 (2022).
49. Liu, Q. *et al.* The histone methyltransferase SUVR2 promotes DSB repair via chromatin remodeling and liquid-liquid phase separation. *Mol. Plant* (2022) doi:10.1016/j.molp.2022.05.007.
50. Shepherd, M. J., Horton, J. S. & Taylor, T. B. A near-deterministic mutational hotspot in Pseudomonas fluorescens is constructed by multiple interacting genomic features. *Mol. Biol. Evol.* (2022) doi:10.1093/molbev/msac132.
51. González-Bermúdez, L., Genescà, A., Terradas, M. & Martín, M. Role of H4K16 acetylation in 53BP1 recruitment to double-strand break sites in in vitro aged cells. *Biogerontology* (2022) doi:10.1007/s10522-022-09979-6.
52. Cañas, J. C. *et al.* A role for the Saccharomyces cerevisiae Rtt109 histone acetyltransferase in R-loop homeostasis and associated genome instability. *Genetics* (2022) doi:10.1093/genetics/iyac108.
53. Weiss, T. *et al.* Epigenetic features drastically impact CRISPR-Cas9 efficacy in plants. *Plant Physiol.* (2022) doi:10.1093/plphys/kiac285.
54. Boukas, L., Razi, A., Bjornsson, H. T. & Hansen, K. D. Natural selection acts on epigenetic marks. *bioRxiv* 2020.07.04.187880 (2022) doi:10.1101/2020.07.04.187880.
55. Fang, J. *et al.* Cancer-driving H3G34V/R/D mutations block H3K36 methylation and H3K36me3-MutSα interaction. *Proc. Natl. Acad. Sci. U. S. A.* **115**, 9598–9603 (2018).

56. Xiao, C. *et al.* H3K36 trimethylation-mediated biological functions in cancer. *Clin. Epigenetics* **13**, 199 (2021).

57. Li, G. *et al.* The Sequences of 1504 Mutants in the Model Rice Variety Kitaake Facilitate Rapid Functional Genomic Studies. *Plant Cell* **29**, 1218–1231 (2017).

58. Weiss, T. *et al.* Drastic differential CRISPR-Cas9 induced mutagenesis influenced by DNA methylation and chromatin features. *bioRxiv* 2022.02.28.482333 (2022) doi:10.1101/2022.02.28.482333.

59. Schep, R. *et al.* Impact of chromatin context on Cas9-induced DNA double-strand break repair pathway balance. *Mol. Cell* **81**, 2216–2230.e10 (2021).

60. Ma, X. *et al.* Mutation hot spots in yeast caused by long-range clustering of homopolymeric sequences. *Cell Rep.* **1**, 36–42 (2012).

61. Lujan, S. A., Clark, A. B. & Kunkel, T. A. Differences in genome-wide repeat sequence instability conferred by proofreading and mismatch repair defects. *Nucleic Acids Res.* **43**, 4067–4074 (2015).

62. Meier, B. *et al.* Mutational signatures of DNA mismatch repair deficiency in C. elegans and human cancers. *Genome Res.* **28**, 666–675 (2018).

63. Hung, S. *et al.* Mismatch repair-signature mutations activate gene enhancers across human colorectal cancer epigenomes. *Elife* **8**, (2019).

64. Tran, H. T., Keen, J. D., Kricker, M., Resnick, M. A. & Gordenin, D. A. Hypermutability of homonucleotide runs in mismatch repair and DNA polymerase proofreading yeast mutants. *Mol. Cell. Biol.* **17**, 2859–2865 (1997).

65. Gragg, H., Harfe, B. D. & Jinks-Robertson, S. Base composition of mononucleotide runs affects DNA polymerase slippage and removal of frameshift intermediates by mismatch repair in Saccharomyces cerevisiae. *Mol. Cell. Biol.* **22**, 8756–8762 (2002).

66. Wang, L. *et al.* The architecture of intra-organism mutation rate variation in plants. *PLoS Biol.* **17**, e3000191 (2019).

67. Monroe, J. G. *et al.* Author Correction: Mutation bias reflects natural selection in Arabidopsis thaliana. *Nature* doi:10.1038/s41586-023-06387-9 (2023).
68. Stoler, N. & Nekrutenko, A. Sequencing error profiles of Illumina sequencing instruments. *NAR Genom Bioinform* **3**, lqab019 (2021).
69. Exposito-Alonso, M. *et al.* The rate and potential relevance of new mutations in a colonizing plant lineage. *PLoS Genet.* **14**, e1007155 (2018).
70. Kim, S. *et al.* Strelka2: fast and accurate calling of germline and somatic variants. *Nat. Methods* **15**, 591–594 (2018).
71. Sloan, D. B., Broz, A. K., Sharbrough, J. & Wu, Z. Detecting Rare Mutations and DNA Damage with Sequencing-Based Methods. *Trends Biotechnol.* **36**, 729–740 (2018).
72. Schmitt, M. W. *et al.* Detection of ultra-rare mutations by next-generation sequencing. *Proc. Natl. Acad. Sci. U. S. A.* **109**, 14508–14513 (2012).
73. Martincorena, I. *et al.* Tumor evolution. High burden and pervasive positive selection of somatic mutations in normal human skin. *Science* **348**, 880–886 (2015).
74. Adamek, K., Jones, A. M. P. & Torkamaneh, D. Accumulation of somatic mutations leads to genetic mosaicism in cannabis. *Plant Genome* **15**, e20169 (2022).
75. Kovalchuk, I., Kovalchuk, O. & Hohn, B. Genome-wide variation of the somatic mutation frequency in transgenic plants. *EMBO J.* **19**, 4431–4438 (2000).
76. Oztas, O., Selby, C. P., Sancar, A. & Adebali, O. Genome-wide excision repair in Arabidopsis is coupled to transcription and reflects circadian gene expression patterns. *Nat. Commun.* **9**, 1503 (2018).
77. Kaya, S., Adebali, O., Oztas, O. & Sancar, A. Genome-wide Excision Repair Map of Cyclobutane Pyrimidine Dimers in Arabidopsis and the Roles of CSA1 and CSA2 Proteins in Transcription-coupled Repair. *Photochem. Photobiol.* **98**, 707–712 (2022).
