# Supplementary material for: Reply to: Re-evaluating evidence for adaptive mutation rate variation
Source: Nature. 2023 Jul 26;619(7971):E57–60. doi: 10.1038/s41586-023-06315-x (PMC10371858; doi:10.1038/s41586-023-06315-x)
Supplement: Supplementary file 2 — Reporting Summary [file 41586_2023_6315_MOESM2_ESM.pdf]

nature portfolio

Corresponding author(s): Grey Monroe
Detlef Weigel

Last updated by author(s): Apr 28, 2023

Reporting Summary

Nature Portfolio wishes to improve the reproducibility of the work that we publish. This form provides structure for consistency and transparency in reporting. For further information on Nature Portfolio policies, see our Editorial Policies and the Editorial Policy Checklist.

Statistics

For all statistical analyses, confirm that the following items are present in the figure legend, table legend, main text, or Methods section.

n/a	Confirmed	
☐	☒	The exact sample size (*n*) for each experimental group/condition, given as a discrete number and unit of measurement
☒	☐	A statement on whether measurements were taken from distinct samples or whether the same sample was measured repeatedly
☐	☒	The statistical test(s) used AND whether they are one- or two-sided<br>*Only common tests should be described solely by name; describe more complex techniques in the Methods section.*
☐	☒	A description of all covariates tested
☐	☒	A description of any assumptions or corrections, such as tests of normality and adjustment for multiple comparisons
☐	☒	A full description of the statistical parameters including central tendency (e.g. means) or other basic estimates (e.g. regression coefficient) AND variation (e.g. standard deviation) or associated estimates of uncertainty (e.g. confidence intervals)
☐	☒	For null hypothesis testing, the test statistic (e.g. *F*, *t*, *r*) with confidence intervals, effect sizes, degrees of freedom and *P* value noted<br>*Give P values as exact values whenever suitable.*
☒	☐	For Bayesian analysis, information on the choice of priors and Markov chain Monte Carlo settings
☒	☐	For hierarchical and complex designs, identification of the appropriate level for tests and full reporting of outcomes
☒	☐	Estimates of effect sizes (e.g. Cohen's *d*, Pearson's *r*), indicating how they were calculated

Our web collection on statistics for biologists contains articles on many of the points above.

Software and code

Policy information about availability of computer code

Data collection	GATK V4, bwa 0.7, Strelka2 2.9.10
Data analysis	Analyses were conducted with R version 4.2.2, github.com/greymonroe/mutation_bias_analysis2, github.com/greymonroe/polymorphology

For manuscripts utilizing custom algorithms or software that are central to the research but not yet described in published literature, software must be made available to editors and reviewers. We strongly encourage code deposition in a community repository (e.g. GitHub). See the Nature Portfolio guidelines for submitting code & software for further information.

Data

Policy information about availability of data

All manuscripts must include a data availability statement. This statement should provide the following information, where applicable:

- Accession codes, unique identifiers, or web links for publicly available datasets
- A description of any restrictions on data availability
- For clinical datasets or third party data, please ensure that the statement adheres to our policy

The TAIR10 A. thaliana reference genome is found at Arabidopsis.org/download. The more recent improved A. thaliana reference genome is found at https://github.com/schatzlab/Col-CEN. Sequencing reads for 107 A. thaliana mutation accumulation lines are stored in the NCBI Short Read Archive (SRA) accession number SRP133100. Additional mutation datasets were downloaded from publications cited in Supplemental Table 1.

Human research participants

Policy information about studies involving human research participants and Sex and Gender in Research.

Reporting on sex and gender	N/A
Population characteristics	N/A
Recruitment	N/A
Ethics oversight	N/A

Note that full information on the approval of the study protocol must also be provided in the manuscript.

Field-specific reporting

Please select the one below that is the best fit for your research. If you are not sure, read the appropriate sections before making your selection.

☒ Life sciences ☐ Behavioural & social sciences ☐ Ecological, evolutionary & environmental sciences

For a reference copy of the document with all sections, see nature.com/documents/nr-reporting-summary-flat.pdf

Life sciences study design

All studies must disclose on these points even when the disclosure is negative.

Sample size	Sample sizes (number of studies examined) were based on the total published literature reporting mutation rates in Arabidopsis thaliana. Citations of these publications are found in Supplemental Table 1.
Data exclusions	Data were filtered according to descriptions in the manuscript. For example, variants were filtered to remove cases of being potentially caused by homopolymer bleed-through.
Replication	We tested whether mutation biases reported were replicated by 10 independent mutation accumulation datasets. In all cases, there was an observation of lower gene body mutation rates, with the exception of MSH2 knockout lines (Belfield et al. 2018) which indicates that MSH2 preferentially repairs gene bodies.
Randomization	NA: Data were observational measures of mutation rate variation across the genome from mutation accumulation experiments.
Blinding	NA: Data were observational measures of mutation rate variation across the genome from mutation accumulation experiments.

Reporting for specific materials, systems and methods

We require information from authors about some types of materials, experimental systems and methods used in many studies. Here, indicate whether each material, system or method listed is relevant to your study. If you are not sure if a list item applies to your research, read the appropriate section before selecting a response.

Materials & experimental systems

n/a	Involved in the study	
☒	☐	Antibodies
☒	☐	Eukaryotic cell lines
☒	☐	Palaeontology and archaeology
☒	☐	Animals and other organisms
☒	☐	Clinical data
☒	☐	Dual use research of concern

Methods

n/a	Involved in the study	
☒	☐	ChIP-seq
☒	☐	Flow cytometry
☒	☐	MRI-based neuroimaging
